# National trends and determinants of diabetes mellitus in turkish adults, 2008–2022

**DOI:** 10.1007/s12020-026-04605-8

**Published:** 2026-04-02

**Authors:** Duha Yahya, Ridvan Sakir, Mehmet Kocak

**Affiliations:** 1 International School of Medicine, Istanbul Medipol University, Istanbul, Türkiye; 2 Basaksehir Cam ve Sakura City Hospital, Istanbul, Türkiye; 3 Health Sciences and Technologies Research Institute (SABITA), Istanbul Medipol University, Istanbul, Türkiye; 4 Department of Biostatistics and Medical Informatics, International School of Medicine, Istanbul Medipol University, Istanbul, Türkiye

**Keywords:** Diabetes mellitus, prevalence, risk factors, obesity, physical activity, logistic regression

## Abstract

**Purpose:**

Diabetes mellitus (DM) is an escalating global health problem, and Turkiye is experiencing a particularly rapid rise in prevalence. Understanding national trends and associated risk factors is essential for developing targeted prevention and disease management strategies. This study aimed to evaluate the prevalence of diabetes in Turkiye and identify associated demographic, anthropometric, behavioral, and familial risk factors using nationally representative surveys over 2008 to 2022.

**Methods:**

This cross-sectional analysis utilized pooled data from seven cycles of the Turkish Health Survey (2008, 2010, 2012, 2014, 2016, 2019, and 2022), comprising 120,044 adult participants. Diabetes status was self-reported. Univariable and multivariable logistic regression models were constructed for adults participants to investigate the association of diabetes likelihood with independent predictors, including age, gender, BMI, waist circumference, education, employment, physical activity, geographic region, and family history of diabetes.

**Results:**

Diabetes prevalence increased significantly over time, from 6.6% in 2008 to 13.2% in 2022. Higher age, female gender, greater BMI and waist circumference, lower education level, and unemployment were independently associated with increased odds of diabetes. Physical activity—both moderate and vigorous—demonstrated reduced likelihood of DM, particularly with higher frequency. Regional variation was evident, with the Aegean and Middle Anatolia regions showing higher prevalence and Northeastern Anatolia the lowest. Having a diabetic parent was associated with an 89% increase in odds, while having a non-diabetic spouse was associated with a 32% reduction.

**Conclusion:**

Diabetes prevalence in Turkiye has nearly doubled over a time-span of 14 years and is associated with a range of modifiable sociodemographic and lifestyle factors. The findings highlight the need for nationwide, regionally fine-tuned, prevention and management programs targeting obesity, sedentary behavior, and social determinants of health, taking also into account the family history.

**Supplementary Information:**

The online version contains supplementary material available at 10.1007/s12020-026-04605-8.

## Introduction

Diabetes Mellitus (DM) refers to a group of metabolic disorders characterized primarily by chronic hyperglycemia, resulting from impairments in insulin secretion, insulin action, or both. The condition can lead to serious complications affecting the cardiovascular, renal, ocular, and nervous systems, contributing significantly to global morbidity and mortality [1, 2]. In recent decades, diabetes has emerged as a major global public health challenge. According to the International Diabetes Federation (IDF), 537 million people were living with diabetes in 2021, accounting for 10.5% of the world’s adult population. This burden contributed to global healthcare expenditures of approximately $966 billion, a figure expected to exceed $1,054 billion by 2045 [3, 4]. Diabetes was the direct cause of 1.6 million deaths in 2021, and nearly half of these deaths occurred before age 70. Moreover, 11% of cardiovascular deaths and over half a million kidney-related deaths were attributable to hyperglycemia [5].

In Turkiye, diabetes represents an escalating burden on both individuals and the healthcare system. In 2021, an estimated 16.2% of adults aged 20–79 were living with diabetes—a rate projected to rise further due to increasing obesity and an aging population [6]. The direct costs of diabetes, including hospitalizations, medications, and treatment of complications (e.g., cardiovascular and renal diseases), were estimated to exceed $9 billion annually [7]. Indirect costs such as reduced productivity, early retirement, and premature death further compound the national economic impact [8]. A substantial proportion of undiagnosed diabetes and prediabetic conditions continues to delay intervention and exacerbate complications later on the life span.

Despite recognition of these burdens, relatively few studies in Turkiye have assessed long-term population-level data using robust statistical models to quantify changing trends and risk factors for diabetes. In this study, we analyzed seven waves of the Turkish Health Survey from 2008 to 2022, revealing a striking increase in diabetes prevalence—from 6.6% in 2008 to 13.2% in 2022. This rise occurred in parallel with broader demographic and lifestyle changes such as population aging, increased urbanization, rising obesity, and declining physical activity.

Aging, high body mass index (BMI), central obesity, low physical activity, lower education level, and family history are established contributors to diabetes risk. However, few national studies have explored these variables over time or applied multivariable logistic regression models to identify independent predictors with their added contributions to the known factors. Additionally, investigations around the association with regional and familial patterns such as spousal or parental diabetes are still lacking.

Therefore, the present cross-sectional national survey series aims to assess both the temporal trends at the national level and the independent risk factors for diabetes in the Turkish population using univariable and multivariable logistic regression models. By evaluating a large, nationally representative dataset spanning 14 years, this study seeks to inform future health policy and guide targeted prevention strategies, specific to Turkiye, but generalizable to any other regional setting.

## Methods and materials

Turkish Health Survey (THS) was first conducted in 2008 and subsequently repeated in 2010, 2012, 2014, 2016, 2019, and 2022 by the Turkish Statistical Institute (TSI). It was designed in alignment with the European Health Interview Survey (EHIS) developed by Eurostat and implemented as part of EHIS waves in Turkiye.

The sampling method has been described in prior publications [9,10], [11]. In brief, the survey employed a stratified two-stage cluster sampling strategy. External stratification was based on distinguishing rural from urban areas. During the first stage, clusters (or blocks) were randomly selected with probabilities proportional to their size. In the second stage, household addresses were systematically and randomly selected from each chosen block. Sampling was conducted across all regions of Turkiye, targeting the general population, with the exception of individuals in institutional living arrangements—such as those in military quarters, dormitories, long-term hospitals, or elder care facilities—and residents of very small settlements that could not yield an adequate number of sample households (e.g., hamlets). The number of households surveyed ranged from 7,910 in 2008 to 11,179 in 2022. Data were collected through face-to-face interviews. The initial round of data collection took place in April 2008. Later survey rounds were carried out in May–June (2010, 2012), August–October (2014, 2016), and September–December (2019, 2022). All survey waves were representative at the NUTS-1 level, covering 12 major socio-economic regions of the country. The participant characteristics were provided in Table A1 by year.

The overall purpose of the THS was to systematically collect information on socio-demographic characteristics, health behavior, healthcare access and utilization, and various health indicators in Turkiye, allowing for longitudinal monitoring of the nation’s health profile and its determinants. According to Eurostat standards, the survey consistently included individuals aged 0–14 and those 15 years and older, though specific question wording occasionally evolved. For participants aged 15 and above, the survey was divided into three primary modules: health status, healthcare services, and health-related behaviors. The health status module included chronic conditions, injuries, sensory and physical impairments, pain, difficulties in daily activities, and mental health concerns. The healthcare services module assessed the use of outpatient and inpatient care, medication intake, and preventive services. The third module, covering health determinants, collected information on weight, height, diet, physical activity, alcohol consumption, and tobacco use.

Participants self-reported their chronic diseases by responding “Yes,” “No,” “Do not know,” or “Refuse to answer.” They could also choose not to respond to specific items. For diabetes melitus (DM), the specific question was “During the past 12 months, have you had diabetes?”. The question did not have any specification regarding gestational diabetes, and the responses are purely Participant Reported Outcome (PRO). For the modelling, only responses of “Yes” or “No” were retained for analysis and used to define the binary outcome variable. Those with missing responses or who selected “Do not know” or “Refuse to answer” were excluded from the analysis. For years 2008, 2010, and 2012, there was an addition question regarding whether or not “diabetes was diagnosed by a medical doctor?”. Respectively for years 2008, 2010, and 2012, 100%, 98.5%, and 99% of participant-reported diabetes cases were reported to be diagnosed by a doctor. Therefore, when combining diabetes outcome over the seven waves of the survey, we sticked to the above diabetes question. As part of the inclusion and exclusion approaches, we included all participants who are 20 years and older and had diabetes mellitus questions answered. We did not exclude anyone based on other comorbidities.

Regions were categorized using the NUTS-1 classification, which divides Turkiye into large geographic and economic zones. Educational attainment was defined based on the ISCED 2011 classification system and Eurostat criteria, and grouped into three levels: less than high school, high school graduate, and college or higher. BMI was categorized into four groups: underweight (< 18.5), normal weight (18.5–24.9), overweight (25.0–29.9), and obese (30.0 and above).

Due to the family-based nature of the survey and the resulting dataset, information on chronic conditions was also available for spouses and the respondent’s parents. For the spousal effect, we generated an indicator showing whether or not the spouse had DM. Similarly for parents, we generated an indicator showing whether or not the parents regardless of gender had DM. We used this feature to identify familial occurrences of the same condition and constructed separate statistical models to evaluate how these family-level variables were associated with the respondent’s likelihood of disease.

For the initial screening of variables, we conducted univariate analyses using Chi-square tests for categorical variables and Wilcoxon-Mann-Whitney tests for continuous variables. Multivariable logistic regression models were then built to assess the relationship between DM likelihood and a range of factors, including year, region, demographic characteristics, body measurements, lifestyle factors, and other health indicators. As moderate and vigorous physical activity measures are expected to be correlated, we chose to use the moderate physical activity level as a predictor in the multivariable models. BMI was missing for 4.8% of the participants and therefore, BMI was not included in the multivariable models. Since these analyses were exploratory in nature, a p-value threshold of < 0.05 was used to determine statistical significance. As the microdata we were given did not have the survey weights, we were not able to construct Weighted Logistic Regression models. All statistical analyses and graphical representations were performed using SAS^®^ Version 9.4 (Cary, North Carolina, USA).

## Results

Among a total of 120,044 participants, 12,387 (10.3%) were diagnosed with diabetes mellitus (DM) (Table 1). The prevalence of DM showed a statistically significant upward trend across the years from 2008 to 2022. In 2008, the proportion of participants with DM was only 6.6% (*n* = 857), increasing to 7.5% in 2010 (*n* = 963), 8.2% in 2012 (*n* = 2039), 11.5% in 2014 (*n* = 1984), 11.9% in 2016 (*n* = 1867), 12.4% in 2019 (*n* = 1949), and reaching 13.2% in 2022 (*n* = 2728). This continuous increase indicates a persistent and accelerating public health concern (Table 1). The increase in DM prevalence is consistent by gender where females have higher likelihood of DM diagnosis (Table 1).

**Fig. 1:**
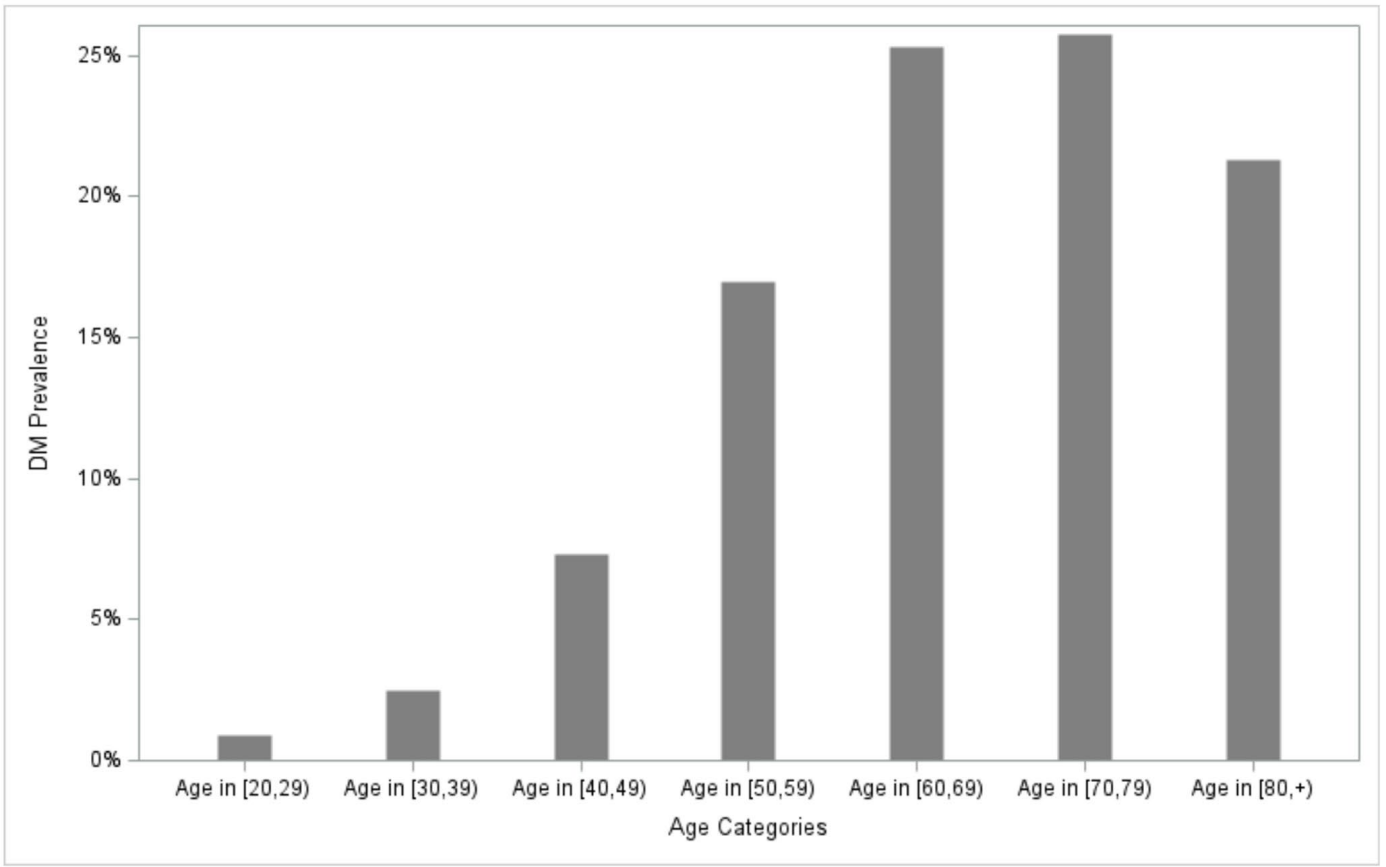
Diabetes Mellitus prevalence by age group

Age had a strong and progressive association with DM prevalence (Fig. 1; Table 1) as anticipated. Only 0.9% of individuals aged 20–29 had DM (*n* = 197), while prevalence increased with each subsequent decade: 2.5% in the 30–39 age group (*n* = 670), 7.3% in the 40–49 age group (*n* = 1839), 17.0% in the 50–59 age group (*n* = 3445), 25.3% in the 60–69 age group (*n* = 3570), 25.7% in 70–79 age group (*n* = 2015), and 21.3% in 80 + age groups (*n* = 651), and this age relationship remains similar between males and females.

Education level showed an inverse association with DM. Participants who had not completed high school had the highest prevalence at 12.7% (*n* = 10,146), while those who completed high school had a significantly lower prevalence of 5.6% (*n* = 1182). Individuals with an undergraduate degree had an even lower rate at 4.7% (*n* = 760), and the DM prevalence for those with graduate-level education bounced back to 8.9% (*n* = 299), possibly due to age-related confounding.

Marital status also had a significant association with DM. Divorced participants had the highest prevalence of DM at 25.6% (*n* = 1574), followed by widowed individuals at 16.1% (*n* = 987), married individuals at 10.5% (*n* = 9552), and singles with the lowest prevalence at 1.6% (*n* = 274), again possibly due to age-related confounding here as well.

Regarding the association of smoking status with DM diagnosis, those who reported regular smoking had a DM prevalence of 11.4% (*n* = 1662), while those who did not smoke regularly had a higher prevalence of 13.2% (*n* = 289). The larger group of non-smokers had a slightly lower prevalence at 10.1% (*n* = 10,436), indicating a complex relationship between smoking status and DM risk, indicating a significant age and smoking status interaction.

**Fig. 2:**
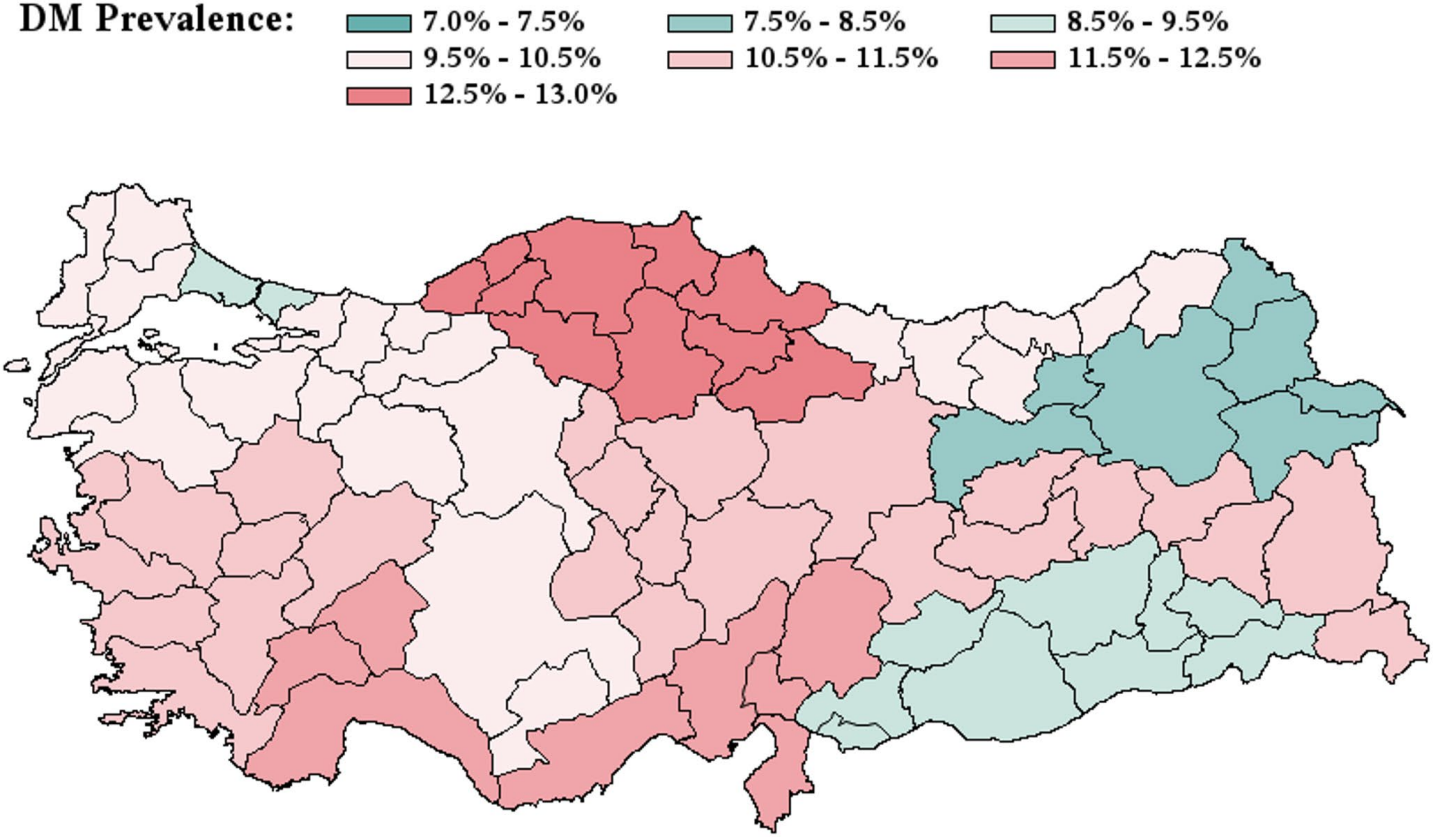
Diabetes Mellitus by Regions of Turkiye

The prevalence of DM exhibited notable regional disparities across Turkiye (Fig. 2; Table 1). The West Black Sea Region reported the highest prevalence at 12.6% (920 out of 7,319participants). The lowest prevalence was recorded in NorthEastern Anatolia at 8.1% (237 of 2,908), emphasizing significant geographic variation in the distribution of DM across the country.

**Table 1:** Diabetes Mellitus Prevalence by Key Factors

Category		Total Participants	With DM (*n*, %)	Without DM (*n*, %)
**All Participants**		120,044	12,387 (10.3%)	107,657 (89.7%)
Age Brackets	Age 20–29	22,704	197 (0.9%)	22,507 (99.1%)
	Age 30–39	26,831	670 (2.5%)	26,161 (97.5%)
	Age 40–49	25,134	1,839 (7.3%)	23,295 (92.7%)
	Age 50–59	20,234	3,445 (17.0%)	16,789 (83.0%)
	Age 60–69	14,628	3,570 (25.3%)	10,558 (74.7%)
	Age 70–79	7,856	2,015 (25.7%)	5,841 (74.3%)
	Age 80+	3,060	651 (21.3%)	2,409 (78.7%)
Marital Status	Single	17,202	274 (1.6%)	16,928 (98.4%)
	Married	90,560	9,552 (10.5%)	81,008 (89.5%)
	Widowed	6,137	987 (16.1%)	5,150 (83.9%)
	Divorced	6,345	1,574 (25.6%)	4,771 (74.4%)
Education Status	< High School	79,592	10,146 (12.7%)	69,446 (87.3%)
	High School	21,874	1,182 (5.6%)	19,742 (94.4%)
	Undergraduate	16,166	760 (4.7%)	15,406 (95.3%)
	Graduate	3,362	299 (8.9%)	3,063 (91.1%)
Smoking Status	Smoking Regularly	14,530	1,662 (11.4%)	12,868 (88.6%)
	Not Smoking Regularly	2,195	289 (13.2%)	1,906 (86.8%)
Region	Aegean Region	17,882	1,967 (11%)	15,915 (89%)
	Central Anatolia Region	6,085	665 (10.9%)	5,420 (89.1%)
	Central East Anatolia Region	5,637	601 (10.7%)	5,036 (89.3%)
	East Black Sea Region	3,408	326 (9.6%)	3,082 (90.4%)
	East Marmara Region	11,181	1,143 (10.2%)	10,038 (89.8%)
	Istanbul Region	23,060	2,156 (9.3%)	20,904 (90.7%)
	Mediterranean Region	15,052	1,754 (11.7%)	13,298 (88.3%)
	Northeast Anatolia Region	2,908	237 (8.1%)	2,671 (91.9%)
	Southeast Anatolia Region	12,006	1,120 (9.3%)	10,886 (90.7%)
	West Anatolia Region	10,458	1,016 (9.7%)	9,442 (90.3%)
	West Black Sea Region	7,319	920 (12.6%)	6,399 (87.4%)
	West Marmara Region	5,048	482 (9.5%)	4,566 (90.5%)

DM varies according to Anthropometric and Lifestyle Characteristics as expected (Table 2). Participants with DM had significantly higher BMI (mean 29.7 vs. 26.3), body weight (79.7 kg vs. 73.4 kg), and waist circumference (96.2 cm vs. 87.3 cm).

In terms of exercise, those without DM reported significantly more frequent moderate physical activity (mean days per week: 0.50 vs. 0.23) and vigorous activity (0.22 vs. 0.07) and walked more often for 10 + minutes (3.80 vs. 3.26 days/week).

**Table 2:** Continuous Predictors of Diabetes Mellitus

	Group	N	Min.	Q1	Median	Q3	Max.	Mean	SD
Height (cm)	Not DM	103,042	52.0	160.0	167.0	173.0	210.0	167.1	8.9
	DM	11,861	120.0	159.0	165.0	170.0	197.0	164.1	8.6
Body Mass Index (BMI)	Not DM	102,503	12.3	23.0	25.8	29.1	66.4	26.3	4.7
	DM	11,834	14.7	25.9	29.2	32.8	64.4	29.7	5.4
Years of Smoking	Not DM	107,657	0.0	0.0	0.0	0.0	80.0	2.3	7.8
	DM	12,387	0.0	0.0	0.0	0.0	72.0	3.5	10.2
No. of Days in a typical week with vigorous exercise	Not DM	106,626	0.0	0.0	0.0	0.0	7.0	0.22	1.1
	DM	12,315	0.0	0.0	0.0	0.0	7.0	0.07	0.64

DM: Diabetes Melitus, SD: Standard Deviation, Q1: First Quartile, Q3: Third Quartile

After adjusting for confounders in two multivariable models, one including all adults > 19 years of age (Model-1, Table 3) and the other including participants > 39 years of age (Model-2, Table 3), several predictors remained consistently significant across both age groups [Table 3]. **DM** prevalence increased significantly **over time**, with 2022 showing nearly double the odds compared to 2008 in both models (OR = 1.96,OR = 1.94 in Models 1 and 2, respectively). **Female gender** was a robust predictor in both models, with higher odds of DM compared to males (OR = 2.36 in Model-1; OR = 1.34 in Model-2). We have confirmed the signifiance of gender through sensitivity analyses by bmi categories and age brackets. For example, in all BMI categories (underweight, normal weight, overweight, and obese), females had consistently higher likelihood of DM controlling for other key predictors. The same conclusion was valid by age categories as well. **Among those who are employed**, the likelihood of DM was significantly lower in both models DM (Table 3). **Greater than High School education** was associated with lower DM likelihood with of OR = 0.74 and 0.73 in Models 1 and 2, respectively. Geographical differences also persisted; participants from **NorthEastern and Western Anatolia** had significantly lower odds of DM compared to Istanbul in both models, indicating persistent regional disparities. Overall, these results highlight consistent sociodemographic and geographic determinants of DM risk across age groups in Turkiye.

**Table 3:** Multivariable Models for Diabetes Mellitus Prevalence in the entire cohort (HS: High School, PA: Physical Activity, CI: Confidence Interval)

Effect	Participants aged 20 + Years[model 1]		Participants aged 40 + Years[Model 2]	
	Odds Ratio (95% CI)	*P*-value	Odds Ratio (95% CI)	*P*-value
Age in [20,29) vs. Age in [40,49)	0.11 (0.10, 0.13)	< 0.0001	NA	NA
Age in [30,39) vs. Age in [40,49)	0.34 (0.31, 0.37)	< 0.0001	NA	NA
Age in [50,59) vs. Age in [40,49)	2.39 (2.25, 2.55)	< 0.0001	2.38 (2.24, 2.54)	< 0.0001
Age in [60,69) vs. Age in [40,49)	3.67 (3.44, 3.92)	< 0.0001	3.64 (3.42, 3.89)	< 0.0001
Age in [70,79) vs. Age in [40,49)	3.70 (3.44, 3.99)	< 0.0001	3.67 (3.40, 3.95)	< 0.0001
Age in [80,+) vs. Age in [40,49)	2.73 (2.46, 3.02)	< 0.0001	2.70 (2.44, 2.99)	< 0.0001
Year 2010 vs. 2008	0.96 (0.87, 1.07)	0.49	0.97 (0.87, 1.07)	0.52
Year 2012 vs. 2008	1.09 (0.99, 1.19)	0.066	1.09 (0.99, 1.19)	0.079
Year 2014 vs. 2008	1.53 (1.40, 1.68)	< 0.0001	1.52 (1.38, 1.67)	< 0.0001
Year 2016 vs. 2008	1.50 (1.37, 1.64)	< 0.0001	1.48 (1.35, 1.63)	< 0.0001
Year 2019 vs. 2008	1.60 (1.46, 1.75)	< 0.0001	1.57 (1.43, 1.73)	< 0.0001
Year 2022 vs. 2008	1.78 (1.63, 1.94)	< 0.0001	1.77 (1.62, 1.94)	< 0.0001
Gender Female vs. Male	1.39 (1.33, 1.45)	< 0.0001	1.36 (1.30, 1.43)	< 0.0001
Education Above HS vs. HS or less	0.74 (0.69, 0.79)	< 0.0001	0.73 (0.68, 0.79)	< 0.0001
Employed? 1: Yes vs. 0: No	0.67 (0.63, 0.70)	< 0.0001	0.65 (0.62, 0.69)	< 0.0001
Aegean vs. Istanbul Region	0.92 (0.86, 0.99)	0.025	0.93 (0.86, 1.00)	0.04
Central Anatolia vs. Istanbul Region	0.97 (0.88, 1.07)	0.54	1.01 (0.91, 1.12)	0.87
Central East Anatolia vs. Istanbul Region	0.98 (0.88, 1.08)	0.63	0.99 (0.89, 1.10)	0.89
East Black Sea vs. Istanbul Region	0.75 (0.66, 0.85)	< 0.0001	0.75 (0.65, 0.86)	< 0.0001
East Marmara vs. Istanbul Region	0.88 (0.81, 0.95)	0.002	0.90 (0.82, 0.97)	0.011
Mediterranean vs. Istanbul Region	1.03 (0.96, 1.11)	0.41	1.06 (0.98, 1.14)	0.12
Northeast Anatolia vs. Istanbul Region	0.65 (0.56, 0.76)	< 0.0001	0.66 (0.57, 0.77)	< 0.0001
Southeast Anatolia vs. Istanbul Region	0.93 (0.86, 1.01)	0.068	0.95 (0.88, 1.04)	0.28
West Anatolia vs. Istanbul Region	0.97 (0.89, 1.05)	0.42	0.98 (0.90, 1.07)	0.71
West Black Sea vs. Istanbul Region	1.04 (0.95, 1.13)	0.39	1.06 (0.97, 1.16)	0.21
West Marmara vs. Istanbul Region	0.75 (0.67, 0.84)	< 0.0001	0.77 (0.69, 0.87)	< 0.0001
No. of Days in a week with Moderate PA	0.96 (0.94, 0.98)	< 0.0001	0.96 (0.94, 0.98)	< 0.0001

## Discussion

This study offers a comprehensive evaluation of the trends and determinants of diabetes mellitus (DM) in Turkiye by analyzing seven waves of a nationally representative national surveys conducted between 2008 and 2022. The results show a substantial and consistent rise in DM prevalence, from 6.6% in 2008 to 13.2% in 2022, reflecting a doubling of the disease burden over 14 years. This trend is consistent with global patterns observed in many middle-income countries experiencing rapid urbanization, changing lifestyles, and population aging [12].

The univariable logistic regression analyses for both the > 19 and > 39 age cohorts identified strong positive associations between DM and several anthropometric and behavioral variables. Specifically, older age, higher BMI, waist circumference, and body weight were significantly associated with increased odds of DM which agrees with literature [13, 14]. Physical inactivity, a well-known risk factor for metabolic diseases, was also consistently linked with higher risk. In contrast, moderate and vigorous physical activity, especially when performed frequently, showed robust negative association with DM likelihood. Even low-frequency activity (e.g., once per week) was beneficial, emphasizing the importance of promoting regular movement [15, 16]. Walking seemed protective only when performed consistently for 5–7 days per week.

The multivariable models allowed for deeper interpretation by viewing the potential impact of all these risk factors in presence of each other. In these models, age remained the most significant independent predictor of DM, with the odds of DM increasing exponentially in older age groups. The year of survey also showed a strong association, independent of other variables, confirming a temporal rise in DM risk across the country. Female gender was associated with significantly higher odds of DM, a finding that may reflect both biological and behavioral differences, as well as gender disparities in healthcare utilization and risk awareness [17].

In agreement with literature, Education emerged as a significant predictor for DM likelihood [18]. Individuals with undergraduate, or graduate education had significantly lower odds of DM compared to those who had high school education or less. This likely reflects differences in health literacy, socioeconomic status, and health-promoting behaviors. Similarly, employment status was inversely associated with DM risk, suggesting that being economically active may be associated with healthier routines, better access to healthcare, and greater awareness of risk factors.

Regionally, there were significant differences in DM prevalence and risk. The West Black Sea Region reported the highest prevalence, while Northeast Anatolia had the lowest. After adjusting for demographic and socioeconomic factors, regions such as Western Anatolia and Eastern Marmara were associated with significantly lower odds of DM compared to Istanbul. These geographic variations may be influenced by factors such as urbanization, dietary habits, healthcare access, and local economic development.

As part of our multivariable models, we also investigated the spousal and parental association. Having a non-diabetic spouse was significantly associated with lower DM risk, which likely reflects shared behaviors and household environments [19, 20]. Moreover, having a diabetic parent nearly doubled the odds of developing DM underscoring the importance of both genetic predisposition and intergenerational lifestyle factors [21, 22].

The strengths of this study are numerous. The use of a large, nationally representative sample collected over multiple survey years with standardized methodology provides a robust foundation for assessing trends and associations. The inclusion of a wide range of sociodemographic, behavioral, and regional variables enables a comprehensive analysis of DM risk. The integration of both univariable and multivariable models strengthens the interpretability of findings and helps differentiate between correlated and independent risk factors. Furthermore, the analysis of familial factors offers novel insights that can inform targeted interventions and screening strategies.

However, the study also has several limitations. Most notably, its cross-sectional design precludes causal inference. While associations are strong and consistent, temporality cannot be established. The reliance on self-reported DM status introduces the potential for underreporting or misclassification, especially among individuals with undiagnosed DM. Additionally, the absence of clinical biomarkers such as fasting glucose or HbA1c levels limits the precision of DM diagnosis. Certain relevant variables, such as dietary intake, DM duration, and medication adherence, were not available, which may affect the completeness of the risk profile. Similarly, smoking behavior indicators were missing for many participants, which limited our multivariable model. Last but not least, Weighted Logistic Regression models were not possible as the As the microdata we were given did not have the survey weights.

Despite these limitations, this study contributes important epidemiological evidence on the evolving burden of DM in Turkiye. The findings highlight several modifiable risk factors—particularly obesity, physical inactivity, and education—that should be central to national prevention strategies. The consistent rise in DM prevalence across regions and over time calls for urgent public health action to reduce risk, prevent complications, and ensure equitable access to care.

## Conclusion

The findings of this large-scale, nationally representative study demonstrate a significant and sustained increase in the prevalence of diabetes mellitus in Turkiye over the 14-year period between 2008 and 2022. This increase—from 6.6% to 13.2%—reflects both global trends and local public health challenges, particularly in the face of rapid demographic aging, increasing obesity, and changing lifestyle patterns.

This study confirmed that age, female gender, higher BMI and waist circumference, unemployment, and lower educational attainment are univariably associated with increased DM risk. Conversely, regular physical activity, higher levels of education, and employment status were associated with significantly lower odds. Regional disparities and the effects of familial clustering (having a diabetic spouse or parent) further highlight the complex interplay of genetic, environmental, and behavioral factors in DM risk.

These findings reinforce the urgent need for targeted public health interventions in Turkiye. Prevention strategies must prioritize the promotion of physical activity, healthy nutrition, early screening, and weight control, particularly among high-risk populations such as older adults, women, the unemployed, and those with lower educational attainment. Additionally, regional policies should address local disparities in access to care and health promotion resources.

Given the cross-sectional design, future longitudinal studies using clinical biomarkers are needed to confirm causality, assess disease progression, and evaluate the long-term impact of interventions. Nevertheless, this study provides a critical epidemiological foundation for guiding national DM control efforts and for designing data-driven, equitable healthcare policies.

## Supplementary Information

Below is the link to the electronic supplementary material.

Supplementary Material 1

## Data Availability

As the death records data utilized in this report were granted access only to the corresponding author, we do not have permission to share these data components; however, we can share the environmental data upon request. Please contact Dr. Mehmet Kocak at [mehmetkocak@medipol.edu.tr](mailto: mehmetkocak@medipol.edu.tr) regarding such data requests.

## Appendix

**Table A1:** Participant Characteristics Across Years

	2008		2010		2012		2014		2016		2019		2022	
	*N*	Col %	*N*	Col %	*N*	Col %	*N*	Col %	*N*	Col %	*N*	Col %	*N*	Col %
Gender (Gender)	5919	45.2	5601	43.5	11,490	45.8	7809	45.2	6907	44.1	7115	45.3	9917	47.9
Male														
Female	7171	54.8	7283	56.5	13,606	54.2	9452	54.8	8748	55.9	8588	54.7	10,808	52.1
agecat	3009	23.0	2493	19.3	4716	18.8	3187	18.5	2724	17.4	2811	17.9	3775	18.2
Age in [20,29)														
Age in [30,39)	3100	23.7	2938	22.8	5925	23.6	3822	22.1	3368	21.5	3367	21.4	4337	20.9
Age in [40,49)	2699	20.6	2791	21.7	5362	21.4	3662	21.2	3166	20.2	3178	20.2	4425	21.4
Age in [50,59)	2019	15.4	2105	16.3	4195	16.7	3027	17.5	2740	17.5	2752	17.5	3558	17.2
Age in [60,69)	1255	9.6	1371	10.6	2665	10.6	2016	11.7	2060	13.2	2061	13.1	2782	13.4
Age in [70,79)	786	6.0	900	7.0	1592	6.3	1062	6.2	1105	7.1	1072	6.8	1362	6.6
Age in [80,+)	222	1.7	286	2.2	641	2.6	485	2.8	492	3.1	462	2.9	486	2.3
education_level	11,196	85.5	11,397	88.5	21,725	86.6	14,661	84.9	13,097	83.7	12,639	80.5	16,121	77.8
High School or less														
Above High School	1894	14.5	1487	11.5	3371	13.4	2600	15.1	2558	16.3	3064	19.5	4604	22.2
Marital Status	1791	13.7	1685	13.1	3545	14.1	2366	13.7	2055	13.1	2276	14.5	3498	16.9
Single														
Married	10,186	77.8	9948	77.2	19,121	76.2	13,083	75.8	11,848	75.7	11,679	74.4	15,001	72.4
Widowed	877	6.7	983	7.6	1809	7.2	515	3.0	530	3.4	574	3.7	892	4.3
Divorced	236	1.8	268	2.1	621	2.5	1297	7.5	1222	7.8	1174	7.5	1334	6.4
Employed?	7847	59.9	7850	60.9	14,977	59.7	10,154	58.8	9401	60.1	9346	59.5	11,844	57.1
No														
Yes	5243	40.1	5034	39.1	10,119	40.3	7107	41.2	6254	39.9	6357	40.5	8881	42.9
NUTS1 Region	1561	11.9	1686	13.1	2991	11.9	2027	11.7	2060	13.2	2021	12.9	2535	12.2
1: Istanbul														
2: Western Marmara	1420	10.8	1396	10.8	2670	10.6	1816	10.5	1625	10.4	1671	10.6	2179	10.5
3: Aegean Region	906	6.9	897	7.0	1596	6.4	924	5.4	921	5.9	892	5.7	1168	5.6
4: Eastern Marmara	671	5.1	757	5.9	1342	5.3	783	4.5	700	4.5	742	4.7	938	4.5
5: Western Anatolia	372	2.8	362	2.8	850	3.4	570	3.3	382	2.4	379	2.4	648	3.1
6: Mediterranean Region	1432	10.9	1255	9.7	2538	10.1	1621	9.4	1542	9.8	1604	10.2	2123	10.2
7: Middle Anatolia	2013	15.4	2069	16.1	3885	15.5	2629	15.2	2350	15.0	2239	14.3	2972	14.3
8: Western Blacksea	976	7.5	1022	7.9	1645	6.6	1199	6.9	1137	7.3	1055	6.7	1808	8.7
9: Eastern Blacksea	2090	16.0	1999	15.5	4962	19.8	3623	21.0	3137	20.0	3327	21.2	3988	19.2
10: North-Eastern Anatolia	346	2.6	318	2.5	571	2.3	388	2.2	344	2.2	337	2.1	469	2.3
11: Middle-Eastern Anatolia	751	5.7	673	5.2	1266	5.0	963	5.6	810	5.2	788	5.0	994	4.8
12: South-Eastern Anatolia	552	4.2	450	3.5	780	3.1	718	4.2	647	4.1	648	4.1	903	4.4
