# Supplementary material for: National trends and determinants of diabetes mellitus in turkish adults, 2008–2022
Source: Endocrine. 2026 Apr 2;91(1):134. doi: 10.1007/s12020-026-04605-8 (PMC13046645; doi:10.1007/s12020-026-04605-8)
Supplement: Supplementary file 1 — Supplementary Material 1 [file 12020_2026_4605_MOESM1_ESM.docx]

APPENDIX

Table A1. Participant Characteristics Across Years

|  | **2008** | | **2010** | | **2012** | | **2014** | | **2016** | | **2019** | | **2022** | |
| --- | --- | --- | --- | --- | --- | --- | --- | --- | --- | --- | --- | --- | --- | --- |
|  | **N** | **Col %** | **N** | **Col %** | **N** | **Col %** | **N** | **Col %** | **N** | **Col %** | **N** | **Col %** | **N** | **Col %** |
| Gender (Gender) | 5919 | 45.2 | 5601 | 43.5 | 11490 | 45.8 | 7809 | 45.2 | 6907 | 44.1 | 7115 | 45.3 | 9917 | 47.9 |
| Male |  |  |  |  |  |  |  |  |  |  |  |  |  |  |
| Female | 7171 | 54.8 | 7283 | 56.5 | 13606 | 54.2 | 9452 | 54.8 | 8748 | 55.9 | 8588 | 54.7 | 10808 | 52.1 |
| agecat | 3009 | 23.0 | 2493 | 19.3 | 4716 | 18.8 | 3187 | 18.5 | 2724 | 17.4 | 2811 | 17.9 | 3775 | 18.2 |
| Age in [20,29) |  |  |  |  |  |  |  |  |  |  |  |  |  |  |
| Age in [30,39) | 3100 | 23.7 | 2938 | 22.8 | 5925 | 23.6 | 3822 | 22.1 | 3368 | 21.5 | 3367 | 21.4 | 4337 | 20.9 |
| Age in [40,49) | 2699 | 20.6 | 2791 | 21.7 | 5362 | 21.4 | 3662 | 21.2 | 3166 | 20.2 | 3178 | 20.2 | 4425 | 21.4 |
| Age in [50,59) | 2019 | 15.4 | 2105 | 16.3 | 4195 | 16.7 | 3027 | 17.5 | 2740 | 17.5 | 2752 | 17.5 | 3558 | 17.2 |
| Age in [60,69) | 1255 | 9.6 | 1371 | 10.6 | 2665 | 10.6 | 2016 | 11.7 | 2060 | 13.2 | 2061 | 13.1 | 2782 | 13.4 |
| Age in [70,79) | 786 | 6.0 | 900 | 7.0 | 1592 | 6.3 | 1062 | 6.2 | 1105 | 7.1 | 1072 | 6.8 | 1362 | 6.6 |
| Age in [80,+) | 222 | 1.7 | 286 | 2.2 | 641 | 2.6 | 485 | 2.8 | 492 | 3.1 | 462 | 2.9 | 486 | 2.3 |
| education_level | 11196 | 85.5 | 11397 | 88.5 | 21725 | 86.6 | 14661 | 84.9 | 13097 | 83.7 | 12639 | 80.5 | 16121 | 77.8 |
| High School or less |  |  |  |  |  |  |  |  |  |  |  |  |  |  |
| Above High School | 1894 | 14.5 | 1487 | 11.5 | 3371 | 13.4 | 2600 | 15.1 | 2558 | 16.3 | 3064 | 19.5 | 4604 | 22.2 |
| Marital Status | 1791 | 13.7 | 1685 | 13.1 | 3545 | 14.1 | 2366 | 13.7 | 2055 | 13.1 | 2276 | 14.5 | 3498 | 16.9 |
| Single |  |  |  |  |  |  |  |  |  |  |  |  |  |  |
| Married | 10186 | 77.8 | 9948 | 77.2 | 19121 | 76.2 | 13083 | 75.8 | 11848 | 75.7 | 11679 | 74.4 | 15001 | 72.4 |
| Widowed | 877 | 6.7 | 983 | 7.6 | 1809 | 7.2 | 515 | 3.0 | 530 | 3.4 | 574 | 3.7 | 892 | 4.3 |
| Divorced | 236 | 1.8 | 268 | 2.1 | 621 | 2.5 | 1297 | 7.5 | 1222 | 7.8 | 1174 | 7.5 | 1334 | 6.4 |
| Employed? | 7847 | 59.9 | 7850 | 60.9 | 14977 | 59.7 | 10154 | 58.8 | 9401 | 60.1 | 9346 | 59.5 | 11844 | 57.1 |
| No |  |  |  |  |  |  |  |  |  |  |  |  |  |  |
| Yes | 5243 | 40.1 | 5034 | 39.1 | 10119 | 40.3 | 7107 | 41.2 | 6254 | 39.9 | 6357 | 40.5 | 8881 | 42.9 |
| NUTS1 Region | 1561 | 11.9 | 1686 | 13.1 | 2991 | 11.9 | 2027 | 11.7 | 2060 | 13.2 | 2021 | 12.9 | 2535 | 12.2 |
| 1: Istanbul |  |  |  |  |  |  |  |  |  |  |  |  |  |  |
| 2: Western Marmara | 1420 | 10.8 | 1396 | 10.8 | 2670 | 10.6 | 1816 | 10.5 | 1625 | 10.4 | 1671 | 10.6 | 2179 | 10.5 |
| 3: Aegean Region | 906 | 6.9 | 897 | 7.0 | 1596 | 6.4 | 924 | 5.4 | 921 | 5.9 | 892 | 5.7 | 1168 | 5.6 |
| 4: Eastern Marmara | 671 | 5.1 | 757 | 5.9 | 1342 | 5.3 | 783 | 4.5 | 700 | 4.5 | 742 | 4.7 | 938 | 4.5 |
| 5: Western Anatolia | 372 | 2.8 | 362 | 2.8 | 850 | 3.4 | 570 | 3.3 | 382 | 2.4 | 379 | 2.4 | 648 | 3.1 |
| 6: Mediterranean Region | 1432 | 10.9 | 1255 | 9.7 | 2538 | 10.1 | 1621 | 9.4 | 1542 | 9.8 | 1604 | 10.2 | 2123 | 10.2 |
| 7: Middle Anatolia | 2013 | 15.4 | 2069 | 16.1 | 3885 | 15.5 | 2629 | 15.2 | 2350 | 15.0 | 2239 | 14.3 | 2972 | 14.3 |
| 8: Western Blacksea | 976 | 7.5 | 1022 | 7.9 | 1645 | 6.6 | 1199 | 6.9 | 1137 | 7.3 | 1055 | 6.7 | 1808 | 8.7 |
| 9: Eastern Blacksea | 2090 | 16.0 | 1999 | 15.5 | 4962 | 19.8 | 3623 | 21.0 | 3137 | 20.0 | 3327 | 21.2 | 3988 | 19.2 |
| 10: North-Eastern Anatolia | 346 | 2.6 | 318 | 2.5 | 571 | 2.3 | 388 | 2.2 | 344 | 2.2 | 337 | 2.1 | 469 | 2.3 |
| 11: Middle-Eastern Anatolia | 751 | 5.7 | 673 | 5.2 | 1266 | 5.0 | 963 | 5.6 | 810 | 5.2 | 788 | 5.0 | 994 | 4.8 |
| 12: South-Eastern Anatolia | 552 | 4.2 | 450 | 3.5 | 780 | 3.1 | 718 | 4.2 | 647 | 4.1 | 648 | 4.1 | 903 | 4.4 |
